# Long-Term Outcomes in Uveal Melanoma After Ruthenium-106 Brachytherapy

**DOI:** 10.3389/fonc.2021.754108

**Published:** 2022-01-03

**Authors:** Gilda Cennamo, Daniela Montorio, Luca D’ Andrea, Antonio Farella, Elide Matano, Mario Giuliano, Raffaele Liuzzi, Maria Angelica Breve, Sabino De Placido, Giovanni Cennamo

**Affiliations:** ^1^ Eye Clinic, Public Health Department, University of Naples “Federico II”, Naples, Italy; ^2^ Department of Neurosciences, Reproductive Sciences and Dentistry, University of Naples “Federico II”, Naples, Italy; ^3^ Radiotherapy Unit, University of Naples “Federico II”, Naples, Italy; ^4^ Department of Clinical Medicine and Surgery, University of Naples Federico II, Naples, Italy; ^5^ Rare Cancer Coordinating Center – Campania Region, Naples, Italy; ^6^ Institute of Biostructure and Bioimaging, National Research Council (CNR), Naples, Italy

**Keywords:** ruthenium-106 brachytherapy, uveal melanoma, survival rate, local recurrence, metastasis, complications

## Abstract

Uveal melanoma is the most common primary intraocular malignancy. The aim of this retrospective study was to report the results after ruthenium-106 (Ru-106) plaque brachytherapy for uveal melanoma in terms of tumor control, visual acuity, radiation-related complications, tumor recurrence, metastases, and patients’ survival rate during 4 years’ follow-up. A total of 355 eyes from 355 patients have been treated with Ru-106 plaque brachytherapy for uveal melanoma between February 2011 and March 2020. Five patients were lost to follow-up, and then 350 eyes of 350 patients (mean age 58 ± 11 years) were enrolled in this retrospective study. All patients underwent a complete ophthalmic examination including echography and spectral domain–optical coherence tomography. The mean follow-up was 4 years (3 months to 9 years). After treatment, the mean tumor thickness was reduced to 1.75 ± 0.21 mm. Radiation complications were found in 63% of patients: 38% showed radiation maculopathy, 11% had optic neuropathy, and 14% developed cataracts. Cancer-free survival was 99%, 97%, and 85%, respectively, at 5, 7, and 9 years. Ru-106 plaque brachytherapy represents a reliable treatment of uveal melanoma. This technique is valid and safe with a low rate of ocular complications during a long-term follow-up.

## 1 Introduction

Uveal melanoma is the most common primary intraocular malignancy, and it represents approximately 5% of all melanomas (1, 2).

Enucleation of the affected eye was the only treatment in the past, but since 1970, the eye-conserving approach has been increasingly used until today (3) in order to preserve vision and the ocular anatomy without increasing the risk of metastatic spread (4).

Today, radiation therapy is the main treatment approach for choroidal melanoma, and the most common irradiation techniques are plaque brachytherapy and proton therapy (5–7). β-Ray source ruthenium-106 (Ru-106) is the most used in Europe (8).

The Collaborative Ocular Melanoma Study (COMS) demonstrated equal melanoma-related survival rates for enucleation and episcleral plaque radiotherapy in medium-sized tumors (measuring 2.5 to 10 mm of apical height and 5 to 16 mm of basal dimension) (4). Furthermore, plaque brachytherapy offers the patient a better quality of life with the possibility to preserve vision (4).

The aim of this retrospective study is to investigate the visual and anatomical outcomes, tumor control, tumor recurrence, distant metastasis, and cancer-free survival in patients undergoing Ru-106 plaque brachytherapy.

## 2 Materials and Methods

### 2.1 Study Design

This retrospective study included all patients with the clinical diagnosis of choroidal melanoma who underwent Ru-106 plaque brachytherapy between February 2011 and March 2020 at the Eye Clinic of the University of Naples “Federico II”.

The gold standard for the diagnosis of choroidal melanoma was based on ophthalmoscopic features and standardized bulbar echography.

A-scan and B-scan ultrasound were performed with an AVISO-S Echograph (Quantel Medical, Clermont-Ferrand, France) and 10- and 20-MHz probes. The axial resolution was 0.2 mm for the A-scan probe, and 0.15 and 0.1 mm for the 10- and 20-MHz B-scan probes, respectively. The dynamic range for B-scan was 25 to 90 dB with adjustable gain until 110 dB.

The tumor size was classified according to the COMS criteria (9, 10) and TNM Staging System (11).

COMS criteria defined small and medium choroidal melanomas as having an apical height of 3 and 3–8 mm, respectively (12).

The inclusion criteria for Ru-106 brachytherapy are patients with small- and medium-size tumors (measuring up to 6.5 mm of apical height) who had at least 3 months of follow-up after treatment.

All patients underwent complete ophthalmic examination, including best-corrected visual acuity (BCVA) according to the Early Treatment of Diabetic Retinopathy Study (ETDRS), slit-lamp biomicroscopy (Haag Streit BM 900), intraocular pressure measurement, fundus biomicroscopy, echography, and spectral domain–optical coherence tomography (SD-OCT) (software RTVue XR Version 2017.1.0.151, Optovue Inc., Fremont, CA, USA).

The screening for distant metastasis was made by liver ultrasonography, chest radiography, and routine blood tests at the time of diagnosis, and these were repeated over time.

The follow-up, including fundus biomicroscopy, echography, and SD-OCT, was performed at 1 month after brachytherapy, at 3-month intervals for 2 years, then twice a year until 5 postoperative years, and then annually. Complications (radiation maculopathy, optic neuropathy, and cataract) were also assessed at each follow-up.

The outcome measures were tumor control, visual acuity, radiation-related complications, tumor recurrence, distant metastases, and cancer-free survival. The Kaplan–Meier survival, performed with the Statistical Package for Social Sciences (Version 25 for Windows; SPSS Inc., Chicago, IL, USA), estimates the probability of survival.

The study adhered to the tenets of the Declaration of Helsinki. Written informed consent was obtained from the patients enrolled in the study. The research protocol was registered on ClinicalTrials.gov (NCT04577742).

### 2.2 Study Techniques

#### 2.2.1 Eye Plaques

Ru-106 ophthalmic plaques CCB and COC types (Eckert & Ziegler BEBIG, Berlin, Germany) were used. The total shell thickness was 1 mm, and it was divided into three layers of thickness, from the inner to outer layer, of 0.1, 0.2, and 0.7 mm. All layers are made of silver, with the middle layer containing the emitter substance. The radioactive nuclide is electrically deposited with an approximate thickness of 0.1 μm on the concave surface. A 0.2-mm-thick silver target foil is sandwiched between the concave surface of a 0.7-mm-thick layer (back) and the convex surface of a 0.1-mm-thick layer (window) (13).

The Ru-106 (half-life 374 days) disintegrates *via* β^−^ decay with a peak beta particle energy of 39 keV to the radioactive daughter Rh-106. The primary contributor to therapeutic dose is the continuous spectrum of beta particles emitted in the decay of Rh-106 (half-life 30 s). Rh-106 disintegrates by β^−^ decay with mean beta energy of about 1.4 MeV and a maximum of 3.5 MeV to the stable element Pd-106.

The 90th percentile distance for Rh-106 beta particles in water is 7.9 mm. Backscatter from the 0.7-mm-thick silver backing of the applicator tends to soften the spectrum (14).

All patients were treated with Ru-106 eye plaque brachytherapy (EPB) to a total dose of 100 Gy to the tumor apex. The time of implant duration was calculated according to the conventional central-axis-point dose calculation (15).

### 2.3 Statistical Analysis

The Kaplan–Meier survival, performed with the Statistical Package for Social Sciences (Version 25 for Windows; SPSS Inc., Chicago, IL, USA), estimates the probability of survival.

## 3 Results

Overall, 355 patients were enrolled of which five patients were excluded because they were lost to follow-up. A total of 350 eyes of 350 patients (200 females and 150 males; mean age 55 years ± 11) were included in the study. The mean follow-up was 4 years (3 months to 9 years).

At baseline, the mean BCVA in affected eyes was 0.32 ± 0.30 logMAR. The mean tumor thickness was 4.52 ± 1.78 mm at A-scan echography. The tumors were classified according to the TNM system as T1 in 220 eyes (63%), T2 in 110 eyes (31%), and T3 in 20 eyes (6%).

According to the COMS system, the tumors were small in 130 eyes (37%) and medium in 220 eyes (63%). The location of the choroidal melanoma was the posterior pole in 119 eyes (34%), between the posterior pole and equator in 175 eyes (50%), and between the equator and ora serrata in 56 eyes (16%). Fifteen eyes presented the tumor in ciliary processes. Patient demographics, tumor, and treatment characteristics are summarized in **Table 1**.

**Table 1:** Demographic, clinical, and ultrasonographic features of 350 eyes with uveal melanoma that underwent Ru-106 brachytherapy.

Eyes (n)	350
**Age mean (range), years**	55 ± 11
**Gender**	
*Male*	150
*Female*	200
**BCVA at baseline (logMar)**	0.35 ± 0.30
**Mean thickness by echography at baseline (mm)**	4.52 ± 1.78
**TNM system (patients n, %)**	
*T1*	220 (63)
*T2*	110 (31)
*T3*	20 (6)
**COMS system (patients n, %)**	
*Small*	130 (37)
*Medium*	260 (63)
**Localization of uveal melanoma (patients n, %)**	
*Posterior pole*	119 (34)
*Posterior pole–equator*	175 (50)
*Equator–ora serrata*	56 (16)
**BCVA posttreatment (logMar)**	0.40 ± 0.25
**Mean thickness by echography posttreatment (mm)**	1.75 ± 0.21
**Complication (patients n, %)**	
*Radiation maculopathy*	135 (38)
*Optic neuropathy*	40 (11)
*Cataract*	50 (14)
**Tumor recurrence (patients n, %)**	3 (1)
**Death due to distant metastasis (patients n, %)**	15 (4)

BCVA, best-corrected visual acuity; COMS, Collaborative Ocular Melanoma Study.

**Figure 1:**
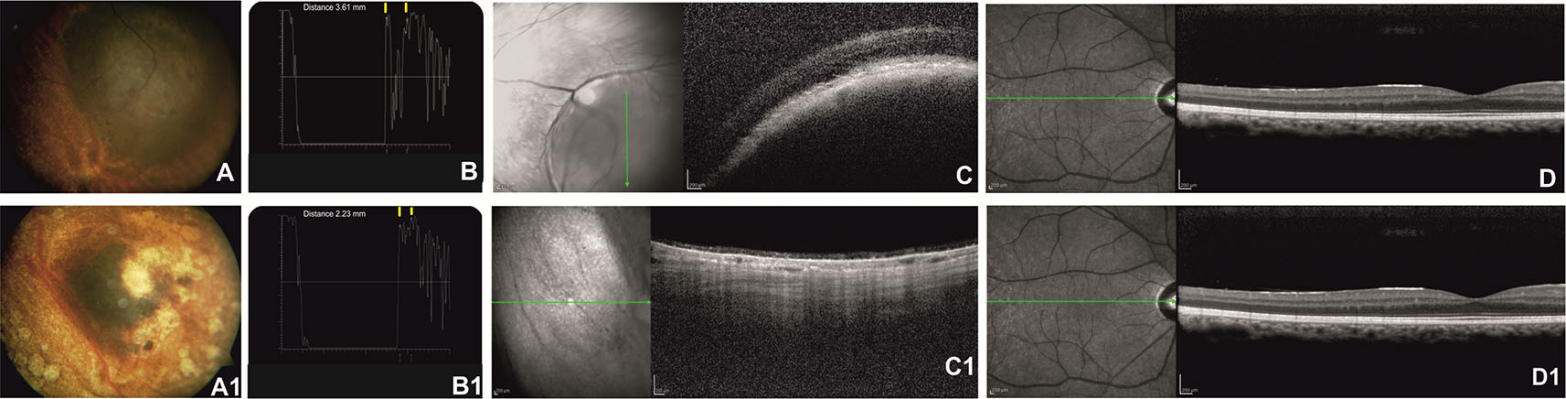
Right eye of a 57-year-old patient affected by choroidal melanoma before ruthenium-106 brachytherapy (top row). Color fundus image shows an elevated and yellow lesion in the nasal mid-peripheral of the retina **(A)**. At A-scan ultrasound, the lesion presents low reflectivity. The yellow arrows over the peaks of the two high and perpendicular spikes, as shown in the echogram, indicate the maximum lift of 3.61 mm **(B)**. Spectral domain–optical coherence tomography (SD-OCT) B-scan over the lesion revealed a highly reflective band within the choriocapillaris layer with posterior shadowing **(C)**. SD-OCT B-scan shows no alteration of the retinal layer architecture in the macular region **(D)**. Color fundus image shows the same tumor after ruthenium-106 brachytherapy **(A1)**. At A-scan ultrasound, the lesion presents high reflectivity with a tumor thickness of 2.23 mm **(B1)** confirmed also by the OCT B-scan **(C1)**. OCT B-scan shows no alteration of the retinal layers architecture in the macular region **(D1)**.

**Figure 2:**
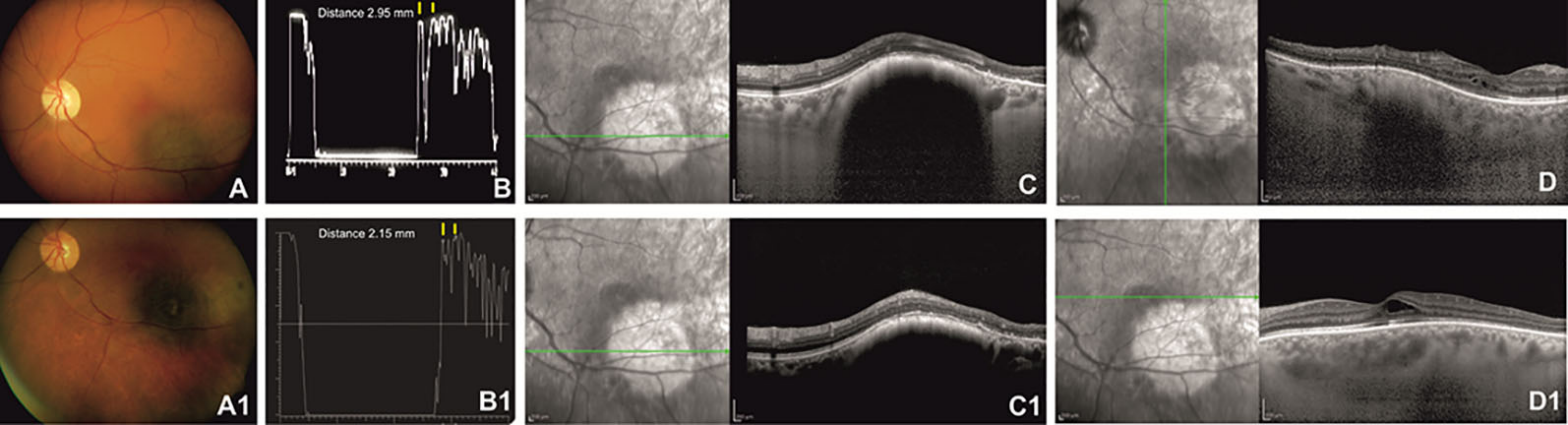
Left eye of a 62-year-old patient affected by choroidal melanoma before ruthenium-106 brachytherapy (top row). Color fundus image shows an elevated and yellow lesion located at the posterior pole **(A)**. A-scan echography shows low reflectivity. The yellow arrows over the peaks of the two high and perpendicular spikes, as shown in the echogram, indicate a tumor thickness of 2.95 mm **(B)**. Spectral domain–optical coherence tomography (SD-OCT) B-scan over the lesion reveals a highly reflective band within the choriocapillaris layer with posterior shadowing **(C)** and normal central retinal thickness with rare intraretinal cysts in the macular region **(D)**. Color fundus image shows the same tumor after ruthenium-106 brachytherapy **(A1)**. At A-scan ultrasound, the lesion presented high reflectivity with a tumor thickness of 2.15 mm **(B1)**, confirmed also by the SD-OCT B-scan **(C1)** that shows in the macular region an increased central foveal thickness with intraretinal cysts due to the radiation maculopathy **(D1)**.

After Ru-106 plaque treatment, the mean tumor thickness was reduced to 1.75 ± 0.21 mm, and the patients who presented radiation-related complications showed a reduced visual acuity (0.7 ± 0.85 logMAR) (**Figures 1** and **2**).

Regarding the complications, 5 years after treatment, 135 patients (38%) showed radiation maculopathy, 40 patients (11%) had optic neuropathy, and 50 patients (14%) developed cataracts.

**Figure 3:**
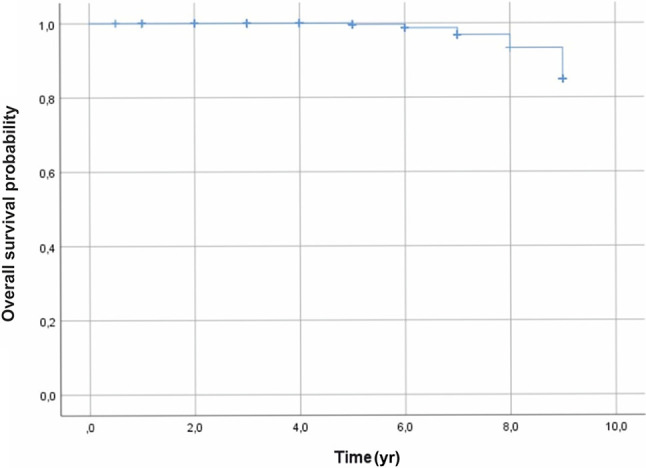
Overall survival outcomes, using the Kaplan–Meier analysis, were 99%, 97%, and 85%, at 5, 7, and 9 years, respectively.

Tumor recurrence was found in three patients at 3 years after the treatment. A total of fifteen deaths occurred due to metastasis from the liver 5 years after brachytherapy. Lastly, the survival rate, using the Kaplan–Meier analysis, was 99%, 97%, and 85% at 5, 7, and 9 years, respectively (**Figure 3**).

## 4 Discussion

Brachytherapy and proton beam radiotherapy are commonly applied in the treatment of uveal melanoma, and they represent the main conservative treatments of choice for patients with choroidal melanoma, showing high performance in the management of tumor treatment (6, 16, 17).

The efficacy of brachytherapy has been demonstrated in terms of patient survival, ocular preservation, control of the tumor, and distant metastasis (18, 19).

Both iodine-125 and Ru-106 brachytherapy had reached excellent tumor control, as reported by previous studies that demonstrated no significant differences in the risk for tumor progression or lack of regression (20).

Takiar et al. reported that 5-year rates of local control, progression-free survival, and overall survival with Ru-106 were 97%, 94%, and 92%, respectively, while for iodine-125, these values were 83%, 65%, and 80%, respectively. In the patients with tumor apex height ≤5 mm, there was no difference in overall survival; however, progression-free survival was significantly improved with Ru-106 (21).

Moreover, the use of Ru-106 brachytherapy allowed to obtain a reliable tumor control with a rate of more than 95% with reduced ocular side effects (22).

In this retrospective study, we reported the results from 350 patients with uveal melanoma after the application of Ru-106 brachytherapy.

We found a high rate of survival, confirmed by previous studies that showed 82% and 72% of survival rates at 5 and 10 years, respectively, as well as, Perri et al., who reported increased rates of 92%, 85%, and 78% at 5, 10, and 15 years, respectively (23).

The 5-year melanoma-related mortality rate was 6% for small and medium tumors and 26% for large tumors, while at 10 years, the mortality rates for small, medium, and large tumors were 14% and 22%, respectively (24).

Other studies showed a rate of 16% and 14% of mortality at 5 years (25, 26), and the mortality rates were 11.4%, 17%, and 23% at 5, 10, and 15 years, respectively (27).

Previous reports also demonstrated excellent local control, ocular preservation, and a high rate of the treated tumor that responded by a significant decrease in tumor height.

The studies conducted by Kaiserman and Georgopoulos reported a reduction of the tumors after 18–24 months, and the tumor height stabilized on an average value of about 60% of the initial height and about 70% after 36 months (28, 29).

Also, the features at A-scan echography showed a significant increase of the internal reflectivity of the uveal melanoma after Ru-106 brachytherapy from a mean of 30%–40% before therapy to 60%–70% after 2 years (28, 29).

Our results showed a low rate of patients undergoing retreatment due to tumor recurrence 3 years after brachytherapy.

Several studies reported that the rate of tumor recurrence following Ru-106 brachytherapy changed significantly between 3%–4% and 11%–16%, and it occurred as early as a year and as late as 10 years after a good response (19, 27, 30).

Our cases reported a rate of 4% of deaths related to metastasis, confirming the results conducted by Seregard et al., which showed that 19 of 220 (9%) patients with successful treatment of the local tumor died of metastases (25).

The studies conducted by Cho and Marinkovic reported a low rate of deaths due to distant metastasis (31, 32); also Verschueren et al. showed a total of 46 deaths of 430 patients for distant metastases after 5 years’ follow-up (30).

Rouberol et al., during 10 years’ follow-up, described a total of 41 deaths from 213 patients due to hepatic metastases and multiple metastases (19).

Despite the localized distribution of this treatment, complications secondary to radiation such as cataracts and radiation maculopathy were found to be the most frequent after Ru-106 brachytherapy.

The cataract is a consequence of direct irradiation of the lens, mostly when the tumor is localized in the anterior part of the choroid and ciliary body. Its probability of developing was 21%, 27%, and 37% at 2, 3, and 5 years after treatment (33).

Radiation maculopathy, a consequence of DNA damage of the vascular endothelial cells, is occlusive retinal microangiopathy that determines important vascular permeability and non-perfusion retinal areas. The mean time to development is at 12 to 24 months after brachytherapy, affecting more than 40% of patients at 5 years (34, 35).

The treatment with anti-vascular endothelial growth factor intravitreal injections demonstrated retinal structural improvements at SD-OCT, although the retinal vascular network became impaired, as detected by OCT angiography (36–38).

Radiation optic neuropathy is less common than radiation maculopathy, and its rate was 10% and 12% after 2 and 3 years, respectively, while iris neovascularization was detected in 12% of the irradiated eyes after treatment (33). Motility disorders and vitreous hemorrhage occur rarely, and they are often temporary (33, 39).

The conservative treatment allows to preserve not only the ocular structures but also the vision. The functional outcomes in this study were confirmed by several reports that demonstrated the impaired visual acuity mainly in cases that showed radiation-related complications due to a reduced distance between the location of the plaque and the foveal region and the optic nerve head (40, 41). A good functional outcome was found in iris melanoma treatment with Ru-106 brachytherapy on the ocular surface, as demonstrated by Agraval et al. This is shown in patients treated between 1998 and 2016: a vision of 6/9 Snellen or better is maintained in 53% of patients with a tumor control of 100%, no melanoma-related mortality, and a 62% reduction in tumor height, observed on ultrasonography (42).

In conclusion, the review of all our data led to confirm that Ru-106 brachytherapy has an excellent rate of local control of uveal melanoma, good survival, and a high rate of ocular preservation with a relatively low rate of recurrence.

## Data Availability Statement

The original contributions presented in the study are included in the article/supplementary material. Further inquiries can be directed to the corresponding author.

## Ethics Statement

The studies involving human participants were reviewed and approved by Federico II University. The patients/participants provided their written informed consent to participate in this study.

## Author Contributions

GilC, GioC, and SD conceived and designed the study. MB, DM, LD’A, EM, MG, and RL performed the data collection. DM analyzed the data and designed the statistical analysis. GilC, DM, and GioC wrote the manuscript. GilC, DM, and AF revised the manuscript. GioC and SD undertook supervision of the study. All authors have read and agreed to the published version of the manuscript.

## Conflict of Interest

The authors declare that the research was conducted in the absence of any commercial or financial relationships that could be construed as a potential conflict of interest.

## Publisher’s Note

All claims expressed in this article are solely those of the authors and do not necessarily represent those of their affiliated organizations, or those of the publisher, the editors and the reviewers. Any product that may be evaluated in this article, or claim that may be made by its manufacturer, is not guaranteed or endorsed by the publisher.
